# A Chemometric Investigation on the Functional Potential in High Power Ultrasound (HPU) Processed Strawberry Juice Made from Fruits Harvested at two Stages of Ripeness

**DOI:** 10.3390/molecules28010138

**Published:** 2022-12-24

**Authors:** Anica Bebek Markovinović, Predrag Putnik, Paula Bičanić, Dora Brdar, Boris Duralija, Branimir Pavlić, Sanja Milošević, Gabriele Rocchetti, Luigi Lucini, Danijela Bursać Kovačević

**Affiliations:** 1 Faculty of Food Technology and Biotechnology, University of Zagreb, Pierottijeva 6, 10000 Zagreb, Croatia; 2 Department of Food Technology, University North, Trg dr. Žarka Dolinara 1, 48000 Koprivnica, Croatia; 3 Department of Pomology, Division of Horticulture and Landscape Architecture, Faculty of Agriculture, University of Zagreb, Svetošimunska Cesta 25, 10000 Zagreb, Croatia; 4 Faculty of Technology, University of Novi Sad, Blvd. Cara Lazara 1, 21000 Novi Sad, Serbia; 5 Department of Animal Science, Food and Nutrition, Università Cattolica del Sacro Cuore, Via Emilia Parmense 84, 29122 Piacenza, Italy; 6 Department for Sustainable Food Process, Università Cattolica del Sacro Cuore, Via Emilia Parmense 84, 29122 Piacenza, Italy

**Keywords:** strawberry juice, maturity, ultrasound, polyphenols, anthocyanins, processing, functional food

## Abstract

This work aimed to investigate the influence of high-power ultrasound (HPU) technology on the stability of bioactive compounds in strawberry juices obtained from fruits with different stages of ripeness (75% vs. 100%) and stored at 4 °C for 7 days. HPU parameters were amplitude (25, 50, 75, and 100%), pulses (50 vs. 100%) and treatment time (5 vs. 10 min). Amplitude and pulse had a significant effect (*p* ≤ 0.05) on all bioactive compounds except flavonols and hydroxycinnamic acids. The treatment duration of 5 min vs. 10 min had a significant positive impact on the content of anthocyanins, flavonols and condensed tannins, while the opposite was observed for total phenols, whereas no statistically significant effect was observed for hydroxycinnamic acids. The temperature changes during HPU treatment correlated positively with almost all HPU treatment parameters (amplitude, pulse, energy, power, frequency). Optimal parameters of HPU were obtained for temperature changes, where the highest content of a particular group of bioactive compounds was obtained. Results showed that by combining fruits with a certain ripeness and optimal HPU treatment, it would be possible to produce juices with highly preserved bioactive compounds, while HPU technology has prospects for application in functional food products.

## 1. Introduction

Strawberry (*Fragaria x ananassa* Duch.) is a seasonal fruit that is very popular among consumers, as well as its products such as jams, purees and juices [1]. This fruit is rich in various bioactive compounds, especially anthocyanins, phenolic acids and flavonoids, which have many health-promoting properties due to their antioxidant effects [2,3,4]. On the other hand, the increased incidence of certain diseases (e.g., obesity, cardiovascular and neurological diseases, cancer) associated with poor diets and unhealthy lifestyles has made consumers in today’s world more aware of healthy diets. Functional foods such as juices [5] are particularly important as part of a healthy diet [6]. Considering their chemical composition and biological potential, strawberries can be considered a functional food [7]. Therefore, strawberry fruits are used in the production of various functional products such as functional dairy products [8,9], functional gluten-free products [10], functional fermented beverages [11] and products [12], functional marmalade [13], functional jelly candies [14], functional fruit juices [15], functional fruit drinks [16] and nectars [17]. 

Functional strawberry juices are of particular interest as they have been shown to possess anti-inflammatory, cardioprotective, anticancer, antioxidant, genoprotective, and neuroprotective properties [7]. Strawberries also showed promising antiviral properties against various pathogenic viruses [18], which is especially important in the current era of global pandemics and crises. Moreover, the global fruit and vegetable juices market was estimated to reach USD 131.62 billion in 2021 and is projected to grow at a compound annual growth rate (CAGR) of 6.3% from 2022 to 2030. Therefore, the interest in producing and consuming juices is steadily increasing [19].

The most critical parameter for successful strawberry juice production is the selection of the cultivar that best suits the cultivation method and ecological conditions [20,21]. Strawberries are susceptible to transportation, which significantly affects their quality, i.e., physical and chemical parameters, bioactive composition and sensory properties [22]. A recent study has confirmed that strawberries harvested at a lower stage of ripeness (75% red fruits) have better structural characteristics for transport and storage, while their processing into juice has also been shown to be good in terms of maintaining quality compared to strawberry juices obtained from 100% ripe strawberries [23]. When processed into juice, fruits with 75% ripeness showed less color change than fruits with 100% ripeness, and sensory characteristics were similar to fully ripe fruits [23]. Thus, by selecting an appropriate cultivar and fruit ripeness level, as well as a strategy for sustainable processing, strawberries could be an excellent raw material for producing high-quality functional juices and serving as fruit meals for consumers while meeting recommendations for higher daily fruit intake [24].

In industrial production, juices are usually preserved by the conventional pasteurization process to extend their shelf-life. Besides the thermolabile bioactive compounds, high temperatures during pasteurization generally cause chemical alterations in volatile fractions and, consequently, affect strawberry products’ nutritional, biological, and sensory quality [25,26]. Hartmann et al. [27] found a significant loss of vitamin C, anthocyanins, total phenols and antioxidant capacity by heating strawberry juices at 85 °C for 5 s and 15 min. A longer processing time at the same temperature (85 °C) resulted in significantly higher losses of polyphenols and anthocyanins. In addition, heating causes reactions between hexoses with amino acids and the formation of Maillard reaction products, which cause browning of the juice and negatively affect the product’s appearance and overall sensory impression [28]. Therefore, the application of innovative non-thermal technologies has been increasingly explored recently. Chemat et al. [29,30] emphasized that when selecting new process technologies, special attention should be paid to environmentally friendly and sustainable technologies that allow for saving both energy and water while ensuring a safe and high-quality product. One of these new technologies is ultrasound-assisted processing. 

The main phenomenon responsible for the effects of ultrasonic processing on liquid media is acoustic cavitation [31], which causes various physical and chemical changes [30]. Compared to thermal pasteurization, ultrasonic processing of juices has the following main advantages: control of enzymatic browning, better preservation of bioactive compounds, prolonged shelf life and improved physicochemical properties of fruit juices [32]. Studies have shown that High Power Ultrasound (HPU) treatment does not cause significant changes in physicochemical and color parameters of strawberry juice and that HPU treatment contributes to significant improvements in bioactive compounds’ contents (i.e., total phenolic content, ascorbic acid, total anthocyanins) and antioxidant activity of the product depending on the adjusted process parameters [33,34,35,36]. 

Since HPU technology has not yet been used to process fruit of different ripeness levels, it is unknown whether this technology would be suitable for processing strawberry juices from two ripeness levels. A recent study applying High Intensity Pulsed Electric Field (HIPEF) has shown that this technology is suitable for processing strawberry juices from different ripening stages, as it improves the stability of bioactive compounds during shelf life [37]. 

In summary, this study aimed to investigate the influence of HPU treatment parameters (amplitude, pulse, and treatment duration) on the stability of bioactive compounds in strawberry juices from fruits of different ripening stages (75% vs. 100%) during storage at 4 °C for 7 days. The application of chemometrics to evaluate the influence of ultrasonic processing parameters was utilized to find optimal HPU processing conditions in terms of the highest stability of bioactive compounds.

## 2. Results and Discussion

### 2.1. The Use of Chemometrics for the Evaluation of HPU Processing

Untreated juices (control samples) from both ripening stages were compared in terms of soluble solids content (SSC) and pH, as well as total phenolic compounds (TPC), monomeric anthocyanins (ANT), hydroxycinnamic acids (HCA), flavonols (FL) and condensed tannins (CT). The levels of all studied bioactive compounds in the control samples were higher in juice from 100% ripe fruit than in 75% (Table 1). Aubert et al. [38] recorded a 1.6 times higher value of total phenols in fully ripe strawberry fruit than in 75% ripe strawberry fruit. The most represented group of bioactive compounds was CT (81.05%), followed by HCA (14.74%), ANT (13.02%) and finally, FL (3.26%). The average values of the bioactive compounds of the control samples are shown in Table 1.

**Figure 1:**
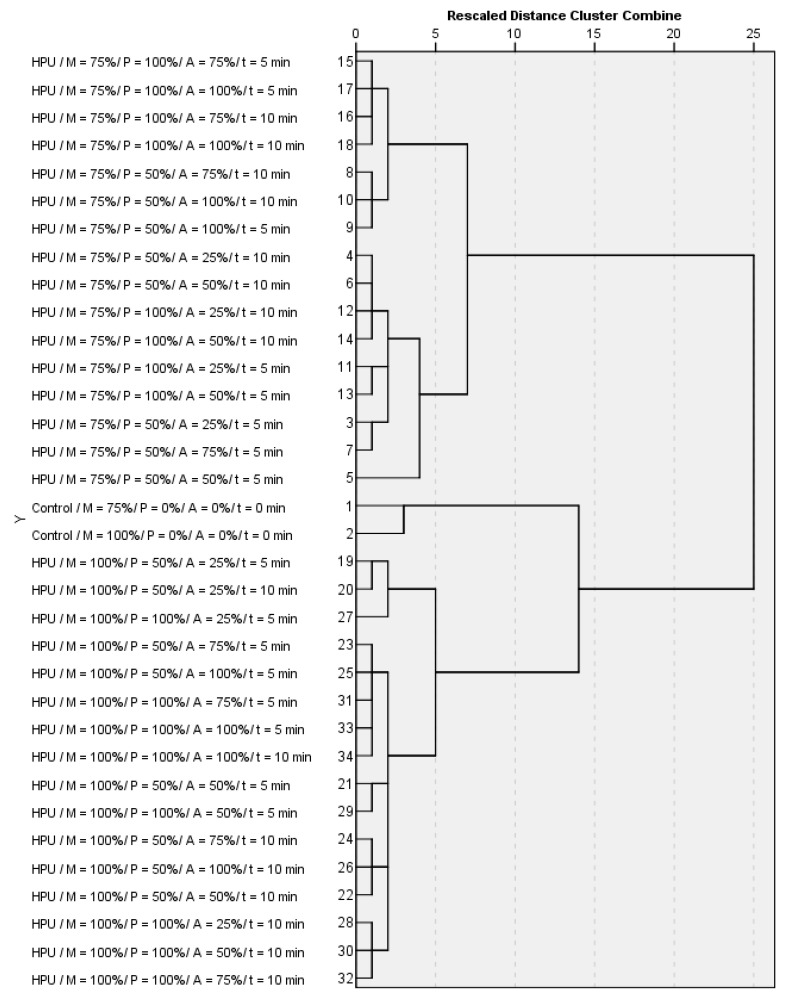
Results of the hierarchical cluster analysis of averaged and standardized samples.

Data analysis initially examined relationships between the samples by an exploratory hierarchical Ward’s cluster analysis. When samples were analyzed for standardized similarities for various types of treatments (controls vs. HPU), maturity (%), HPU pulse (%), amplitude (%), treatment time (min), content of TPC (mg 100 mL^−1^), ANT (mg 100 mL^−1^), HCA (mg 100 mL^−1^), FL (mg 100 mL^−1^), CT (mg 100 mL^−1^), SSC (%), and pH, it was revealed that the most similar samples to controls were those treated with a maturity of 100%, a pulse of 50%, an amplitude of 25%, and treatment durations of 5 and 10 min. They were similar to controls and clustered together regardless of maturity (Figure 1). Other samples similar to controls were those with a maturity of 100%, a pulse of 100%, an amplitude of 25%, and a treatment duration of 5 min.

More detailed examination with Kruskal–Wallis analysis revealed that the chemical profile of the control samples compared with the HPU-treated samples was similar for all polyphenolic groups, except for CT. This group of compounds was found in higher amounts in the HPU samples (Table 2). These results could be a consequence of the effect of cavitation under the influence of HPU [39], which degrades the cellular structure and allows easier CT extraction into the surrounding juice [40,41]. In their research, Bautista-Ortin et al. [42] concluded that HPU treatment is the most effective method to preserve and extract CT during red winemaking, regardless of the different maceration times. Similar results were confirmed by the research of Plaza et al. [43].

### 2.2. The Changes of Bioactive Compounds in Strawberry Juices under HPU Processing and Storage

Table 3 shows the influence of ripeness and storage on the control juice samples.

The levels of all studied bioactive compounds in the control samples, except for total phenolic compounds and FL, were significantly higher (*p* ≤ 0.01) in juices made from 100% ripe fruit than those of 75% ripeness, this is largely consistent with our previous findings [37]. As ripeness increased, TPC decreased in control samples, which is consistent with other literature [44]. Indeed, phenolic compounds are synthesized in the skin of fruits, and considering the lower mass of unripe fruits, their proportion is higher in unripe (smaller) fruits than in larger (riper) fruits [44]. Anthocyanins, the compounds responsible for the red color of strawberries, were almost twice as abundant in juices made of 100% ripe strawberries vs. 75%. Considering that the intensity of the red color increases as strawberries ripen, it was expected that the riper fruits and their products would have higher levels of anthocyanins, which is consistent with the results of Pradas et al. [45]. The HCA content in juices from fully ripe strawberries is almost 52% higher compared to juices from 75% ripe fruits. The higher HCA concentration in the juice of fully ripe strawberries can be explained by the accumulation of phenolic acids [45]. FL was the only exception that was not affected by ripeness. According to studies by Pradas et al. [45], the degree of ripeness had no statistically significant effect on the proportion of flavonols, mainly determined by the cultivar.

Storage positively influenced the contents of HCA and FL in control samples, while the contents of ANT decreased (Table 3). The value of HCA was 6.2% higher after 7 days of storage than after 0 days, which is in agreement with other literature reports [37,38]. The previous study confirmed that total and individual anthocyanins were degraded during storage according to first-order reaction kinetics, and the rate was strongly dependent on temperature [46]. However, storage of the juices for 7 days did not affect the content of TPC and CT. Similarly, the TPC content of the lychee juices after storage at 4 °C for 168 h was not significantly lower than that of the different lychee juices before storage [47].

The changes in bioactive compounds in HPU-treated juice samples during storage are shown in Table 4.

Juices from riper strawberries had higher levels of ANT, HCA, FL, and CT, but the same as in untreated juices, TPC content was higher in the samples from less ripe strawberries (75%). As mentioned earlier, higher TPC contents were found in unripe fruits compared to ripe fruits [44].

To find the optimal parameters for HPU processing of strawberry juices, it is necessary to consider the influence of each HPU process parameter on the stability of bioactive compounds. Thus, increasing the amplitude from 25% to 75% resulted in a statistically significant decrease in the TPC value, while further increasing the amplitude up to 100% significantly increased the TPC value. These results may indicate an optimal amplitude value at which the TPC is best preserved and emphasizes the need to optimize HPU parameters. In the study by Bursać Kovačević et al. [48], increasing the amplitude from 40 to 80% decreased (*p* ≤ 0.05) the TPC value, which is in agreement with our study. On the other hand, Pala et al. [49] reported that amplitude (50%, 75%, and 100%) did not statistically significantly affect the TPC value in HPU-treated pomegranate juice.

Increasing the amplitude from 25 to 100% significantly decreased the content of ANT. Tiwari et al. [50] found the same trend where increasing the amplitude caused a statistically significant decrease in ANT content in HPU-treated strawberry juice (1500 W, 20 kHz). A significant decrease in ANT content at higher amplitudes (75 and 100%) in HPU-treated pomegranate juice was found by Pala et al. [49]. A possible reason for the observed trend could be explained by cavitational collapse and free radical formation [51,52]. Moreover, the increase in amplitude significantly decreased HCA and CT. These results are consistent with the studies of Lukić et al. [53], who found that higher frequencies negatively affected the concentrations of total tannins in red wine. The studies performed by Celotti et al. [54] are in some agreement with the results obtained, as they found the same trend of decreasing CT value with increasing amplitude from 41 to 81% in red wine treated with HPU (200 W, 20 kHz) at 15 and 30 day storage.

Unlike the other groups of compounds, FL was the only exception and was not affected by the variations in amplitude. A similar pattern was found in the research of Bursać Kovačević et al. [48], where variations of amplitude (40 and 80%) did not significantly affect the content of total flavan-3-ols in HPU-treated cloudy apple juice.

As for the pulse, there are two basic types of ultrasound generation, continuous and pulsed. In pulsed generation, the ultrasound output of the generator is switched on and off for a short time and the process is repeated. However, the effect of pulse modulation on the quality characteristics of fruit juices is still not thoroughly explored [32]. Therefore, in this study, we aim to investigate how two pulse modes affect bioactive juice quality. To that end, a higher pulse duration (100%) positively affected the stability of TPC, while higher values of ANT, FL and CT were observed when treated with a lower pulse duration (50%). This influence was not related to the HCA content. In the literature, it is not possible to find a reason for this trend. Still, the 100% pulse may be too invasive for the stability of the studied subgroups of polyphenolic compounds; therefore, for their preservation, HPU treatment with a lower pulse is suggested.

When considering the influence of treatment duration, it appears that a longer duration of sonication (10 min vs. 5 min) has either no effect (TPC and HCA) or a negative effect (ANT, FL, CT). Studies by other authors also confirmed the insignificant effect of treatment time on TPC [48,49,55,56]. Moreover, increasing the pulse from 50 to 100% and the treatment time from 5 to 10 min decreased ANT content. Studies by Tiwari et al. [50] confirmed the significant effect of treatment time on the reduction of ANT in HPU-treated strawberry juices. Wang et al. [35] found that total phenolics increased significantly with the increasing duration of ultrasound treatment. The authors explained this by adding sonochemically generated hydroxyl radicals (OH˙) to the aromatic ring of the phenolic compounds in the ortho-, meta- and para-positions. In addition, they suggested that during ultrasound treatment, the increase in mass transfer rates and the possible disruption of the cell wall of strawberry tissue due to the formation of microcavities could also lead to the release of more phenolic constituents into the juice. Another important effect when strawberry juices are sonicated is the change in the microstructure of the pulp tissue of the juice. The change in cell structures caused by ultrasound treatment increased with increasing treatment time. Therefore, variations regarding the different influence of processing time on different subgroups of polyphenolic compounds are also possible, considering that not all are located at the same positions in the cellular structures [35].

Storage had a statistically significant effect on the reduction of TPC in treated juice after 7 days of storage. These results are in agreement with those of Bursać Kovačević et al. [48], who found the same trend in HPU-treated cloudy apple juice (100 W, 30 kHz frequency) when stored at 4 °C for 7 days.

The 7 day storage had a statistical effect on the decrease of ANT content in the treated juices, which agrees with the results of Tiwari et al. [51], who found a 10% loss of ANT in strawberry juice during 10 day storage at 4 °C. Decreasing ANT levels during the storage of HPU juices has also been reported in other studies [57].

In contrast to TPC and ANT, whose levels decreased after 7 days of storage, HCA and FL increased and the level of CT remained constant. This may indicate that condensation of ANT increases the level of CT while HCA and FL are released into the juices. Similar patterns were previously observed in control samples (Table 3), so it is fairly safe to assume that these changes are due to certain natural processes that occur in strawberry fruit during ripening and storage. In any case, this transformation is not efficient enough to keep TPC constant, so it decreases during storage. In other words, the influences from the HPU samples can be well detected and separated from those of natural occurrences. Tomadoni et al. [34] observed that both control and ultrasound-treated strawberry juices increased phenolic compounds during storage in the refrigerator (0, 3, 7, and 10 days). The authors concluded that the changes that caused senescence and decomposition of the cell structure and, consequently, the release of free phenolic acids and free amino acids, may contribute to the increase in polyphenol contents [34]. Finally, the results on the influence of storage on CT are consistent with the results of Lukić et al. [53], who found no statistically significant influence of storage times of 3 and 6 months on the content of total tannins in HPU-treated red wine (700 W, 20 kHz).

### 2.3. Optimization of HPU Parameters for Strawberry Juice Treatment

Strawberry is an important fruit used for juicing due to its health-promoting bioactive constituents and potential health effects [2]. However, processing and storage conditions may play an important role in the bioavailability of these health-promoting compounds [58]. A previous study suggests that strawberry juices have increased levels of anthocyanins and other phenolic components from strawberry after processing by means of innovative technologies, exhibiting larger amounts compared to the untreated samples. This is suggested to be related to increased matrix disruption and extractability from the matrix, thus liberating bioactive compounds and making them more available for digestion [59]. Therefore, processing parameters and storage conditions must be optimized to ensure the highest quality of stored juices to provide potential health-benefits.

Numerous studies have already been conducted showing the negative influence of temperature on bioactive compounds such as total phenols, anthocyanins, flavonoids, vitamin C and others [27,60,61]. Since temperature changes are crucial for maintaining nutrient content and bioactive value in juices, we wanted to find out how temperature change (ΔT) relates to other HPU parameters. As shown in Table 5, ΔT ranged from 4 to even 54 °C during HPU processing. It is evident that this is a wide temperature range that certainly has an impact on the quality of the treated juices.

From Table 6, it can be seen that ΔT was strongly positively associated with all HPU parameters, namely: energy, power, pulse, amplitudes, and frequency. The only exception was the length of treatment time, which showed the same pattern as the other HPU parameters, except that the correlation was slightly weaker. The increase in amplitude correlated positively (0.59) with temperature change. All other relationships are listed in the same table. In the study of Margean et al. [62], by changing the amplitude parameter from 50 to 70%, an increase in temperature was observed in red grape juice during HPU treatment (750 W, 20 kHz).

Considering that temperature changes correlated with HPU parameters, Table 7 shows the optimal temperature changes (ΔT) and the optimal degree of ripeness at which the maximum content of bioactive compounds is reached. Thus, the highest content of total phenolic compounds (TPC) and FL, respectively, 102.96 mg 100 mL^−1^ and 2.68 mg 100 mL^−1^, can be obtained from fruits with a ripening degree of 75% and a temperature change of 4 °C.

Relating the optimal temperature to the applied HPU treatment parameters (Table 5), it can be observed that a temperature change of 4 °C corresponds to the HPU parameters amplitude 25%, pulse 50%, and treatment duration 10 min, at which the highest values of TPC, FL, and CT were obtained. From this it is clear that higher temperatures do not favor the content of TPC, FL and CT. These results agree with the study of Jabbar et al. [63], where with an increase in temperature from 20 to 60 °C during thermosonication, a significant decrease in the content of TPC, FL and CT in carrot juice was observed. Similar results were obtained by Dundar et al. [64], in which increasing the temperature from 25 to 75 °C during ultrasonication negatively affected the content of total phenols in cloudy strawberry nectar, more specifically, the highest TPC yield of 779.8 mg L^−1^ was obtained at a temperature of 25 °C.

Wahia et al. [65] optimized the thermosonication of orange juice in the temperature range of 45–70 °C and found that the optimal parameters with the highest content of total phenols were 495.34 mg 100 mL^−1^ at a temperature of 49.53 °C, a treatment time of 28.87 min and a frequency of 20.85 kHz. On the other hand, Pokhrel et al. [66] did not observe any statistically significant effect of temperature (50–58 °C) during ultrasound treatment on the content of total phenols in carrot juice.

In contrast to TPC, FL, and CT, the maximum contents of ANT and HCA, 15.58 mg 100 mL^−1^ and 14.14 mg 100 mL^−1^, respectively, were measured at significantly higher temperature changes, 35.41 °C and 36.77 °C, of fully ripe fruit (Table 7). Again, comparing the temperature change with the applied HPU parameters from Table 5 and interpolating, we obtained that the highest values of ANT and HCA were obtained at a pulse of 100% and a treatment time of 5 min with different amplitudes of 70.5% and 88.5%, respectively.

Margean et al. [62] confirmed that the HCA content in red grape juice increases with an increase in amplitude from 50 to 70% as well as temperature. As can be seen from the results, HCA and ANT gave higher yields at higher temperatures. This is confirmed by the studies of Dundar et al. [64] who observed the highest ANT content in cloudy strawberry nectar during ultrasonic treatment at 50 °C.

## 3. Materials and Methods

### 3.1. Chemicals and Standards

HPLC 99% pure methanol obtained from Honeywell (Honeywell, Paris, France) was used as an extraction solvent, while Folin–Ciocalteau reagent obtained from Fisher Scientific (Fisher Scientific, Loughborough, UK) was used for spectrophotometric determination of total phenols. Hydrochloric acid (37%, *w*/*w*), sulfuric acid (96%, p.a.), sodium carbonate, anhydrous (99.5–100.5%), and formic acid (98%, p.a.) were obtained from Lachner (Lachner s.r.o., Neratovice, Czech Republic). Ethanol (96% pure) was obtained from Gram-mol (Gram-mol d.o.o., Zagreb, Croatia). Quercetin (95%) and gallic acid standard (97.5–102.5%) were purchased from Acros Organics (Acros Organics, Guangzhou, China) and Sigma-Aldrich (Sigma-Aldrich Co., St. Louis, MO, USA). Vanillin (99%), potassium chloride (99.0–100.5%), sodium acetate anhydride (99%), and chlorogenic acid (min. 95%) were purchased from Thermo Fisher (Thermo Fisher GmbH, Kandel, Germany).

### 3.2. Production of Strawberry Juice

Strawberry fruits (*Fragaria x ananassa* Duch, cv. ‘Albion’) were grown and harvested in 2021 at Jagodar HB, Donja Lomnica, Croatia. Fruits were harvested at two different ripening stages: (i) 75% ripe fruits, i.e., technological ripening (F1), and (ii) 100% ripe fruits, i.e., consumption ripening stage (F2). After harvest, fruits were delivered to the laboratory, cleaned (stems were removed, washed with tap water and dried with cellulose) and stored in plastic containers at −18 °C until processing. Strawberries were thawed overnight in the refrigerator before processing into juices. Juices (J1 and J2) were prepared from fruits of the corresponding ripeness level (F1 and F2) by cold pressing in a Kuvings B6000 Slow Juicer (VerVita d.o.o., Zagreb, Croatia). The prepared juices were immediately subjected to HPU treatment as described in Section 3.3. All juices were filled into hermetically sealed sterile glass bottles.

### 3.3. High Power Ultrasound (HPU) Processing of Strawberry Juice

Strawberry juice samples were treated with high-power ultrasound on a Hielscher UP400St device (Hielscher Ultrasonics GmbH, Teltow, Germany). The UP400St device consists of a digital ultrasound processor, titanium DN22 (546 mm^2^) sonotrode, a stainless-steel base, and an acrylic glass soundproof box. The maximum power of the UP400St ultrasonic processor is 400 W, the amplitude is adjustable from 20 to 100%, the pulse from 10 to 100%, and the treatment time is set manually in the range from 0.1 s to 99 days. The device also includes a digital thermometer with a range of −50 to 200 °C to measure the temperature of the sample before, during and after treatment.

The control samples were untreated juices, while the HPU samples were treated by varying the HPU parameters: amplitude (25, 50, 75 and 100%), pulse (50 and 100%), and treatment duration (5 and 10 min), according to the experimental design (Table 8). Juices were analyzed immediately after HPU treatment, and after storage at 4 °C for 7 days.

### 3.4. Extraction of Bioactive Compounds

The extraction of bioactive compounds from juices J1 and J2 was prepared according to a modified protocol from the literature [67]. Immediately before extraction, the juice samples were briefly homogenized using a vortex shaker (Grant Instruments Ltd., Cambs, UK). The extraction procedure was performed by pipetting 5 mL of a homogenized sample of strawberry juice into an Erlenmeyer flask and adding 20 mL of the extraction solvent (1% formic acid in 80% methanol, *v*/*v*). Then, the prepared mixture was extracted in an ultrasonic bath DT 514 H Sonorex Digitec 13.5 L (Bandelin electronic GmbH, Berlin, Germany) at 50 °C for 15 min. After extraction, the supernatants were filtered into 25 mL volumetric flasks and made up to the mark with extraction solvent and stored at 4 °C until analysis. All extracts were prepared in duplicates.

### 3.5. Determination of Total Phenolic Content (TPC)

A modified Follin–Ciocalteu method from the literature was used to determine the TPC [68]. Total phenols were determined by pipetting 400 µL of the extract (previously diluted 1:1 with the extraction solvent), 400 µL of the F.C. reagent (previously diluted 5× with distilled water), and 4 mL of a 7.5% sodium carbonate solution. The reaction mixture was allowed to stand at room temperature for 20 min and then the absorbance was measured at 725 nm using a LLG-uniSPEC 2 Spectrophotometer (Lab Logistics Group GmbH, Meckenheim, Germany). The determination for each sample was prepared in parallel. The TPC was calculated from a calibration curve prepared with different concentrations of gallic acid solutions (10–250 mg L^−1^), and results were expressed as mg gallic acid equivalent (GAE) per 100 g or 100 mL of sample.

### 3.6. Determination of Total Monomeric Anthocyanins (ANT)

The spectrophotometric pH differential method has been used to determine ANT [69]. Briefly, 1 mL of the extract was mixed with 4 mL of 0.4 M buffer pH 4.5 (sodium acetate buffer) and separated with 4 mL of 0.025 M buffer pH 1.0 (potassium chloride buffer). After standing for 20 min at room temperature, the absorbance of the reaction mixture was measured at 520 and 700 nm on a LLG-uniSPEC 2 Spectrophotometer (Lab Logistics Group GmbH, Meckenheim, Germany). Determination was prepared in parallel for each sample, and deionized water was used as a blank. According to the equation from the literature [69], the concentration of monomeric anthocyanins is expressed as pelargonidin-3-glucoside equivalent (Pg-3-G) (mg 100 mL^−1^).

### 3.7. Determination of Total Hydroxycinnamic Acids (HCA)

A modified spectrophotometric method described in the literature was used to determine HCA [70]. Briefly, 250 µL of solution 1 (1 g L^−1^ solution of HCl dissolved in 96% ethanol) and 4.55 mL of solution 2 (2 g L^−1^ HCl dissolved in distilled water) were added to 250 µL of the extract. After homogenization for 1 min with a vortex shaker (Grant Instruments Ltd., Cambs, UK), the reaction mixture was allowed to stand in the dark at room temperature for 30 min. Then, the color reaction was measured at 320 nm using a LLG-uniSPEC 2 Spectrophotometer (Lab Logistics Group GmbH, Meckenheim, Germany). For the blank, the determination procedure was identical, except that the extraction solvent was used instead of the extract. For each sample, the measurements were performed in parallel. A calibration curve was prepared from different concentrations of chlorogenic acid solutions (10–600 mg L^−1^), which was used to determine the HCA content in the extracts. The results were expressed as mg chlorogenic acid equivalent (CAE) per 100 g or 100 mL of the sample.

### 3.8. Determination of Total Flavonols (TF)

A modified spectrophotometric method described in the literature was used for the determination of TF [70]. Briefly, 250 µL of solution 1 (1 g L^−1^ HCl dissolved in 96% ethanol) and 4.55 mL of solution 2 (2 g L^−1^ HCl dissolved in distilled water) were added to 250 µL of the extract. After homogenization for 1 min with a vortex shaker (Grant Instruments Ltd., Cambs, UK), the reaction mixture was allowed to stand in the dark at room temperature for 30 min. Then, the color reaction was measured at 360 nm on a LLG-uniSPEC 2 Spectrophotometer (Lab Logistics Group GmbH, Meckenheim, Germany). For the blank, the determination procedure was identical, except that the extraction solvent was used instead of the extract. For each sample, the measurements were performed in parallel. A calibration curve was prepared from different concentrations of the quercetin solution (10–600 mg L^−1^) from which the content of FL in the extracts was determined. The results were expressed as mg quercetin equivalent (QE) per 100 g or 100 mL of the sample.

### 3.9. Determination of Condensed Tannins (CT)

A modified spectrophotometric method described in the literature was used to determine CT [71]. Briefly, 2.5 mL of reagent 1 (25% H_2_SO_4_ solution in methanol) and 1 mL of extract were added to 2.5 mL of reagent 2 (1% vanillin solution in methanol). After homogenization for 1 min with a vortex shaker (Grant Instruments Ltd., Cambs, UK), the reaction mixture was allowed to stand at room temperature for 10 min. The color reaction was then measured at 500 nm using a LLG-uniSPEC 2 Spectrophotometer (Lab Logistics Group GmbH, Meckenheim, Germany). For the blank, the determination procedure was identical, except that the extraction solvent was used instead of the extract. For each sample, measurements were performed in parallel. A calibration curve was generated from different concentrations of catechin solution (10–120 mg L^−1^) and the results were expressed as mg catechin equivalent (CA) per 100 g or 100 mL of the sample.

### 3.10. Statistical Analysis

Descriptive statistics were used for the characterization of the sample. Discrete variables were tested by MANOVA. Exploratory hierarchical Ward’s cluster analysis was used for measuring standardized similarities in samples. Nonparametric analysis employed the Kruskal–Wallis test. Pearson’s linear correlation tested the relation between the pairs of continuous variables. Linear regression was employed to build and compare mathematical models. The level of significance for all tests was α ≤ 0.05, and results were analyzed using SPSS software (v.22). Statgraphics Centurion XVII was used to build and compare mathematical models (Statpoint Technologies Inc., Warrenton, VI, USA).

## 4. Conclusions

HPU technology was used for the first time in producing functional strawberry juices from fruits with different degrees of ripeness. The results showed that strawberry fruit juices treated with HPU from fruits with 100% ripeness had higher contents of ANT, HCA, FL, and CT than juice samples from fruits with 75% ripeness, whereas the opposite trend was observed for TPC. Therefore, strawberry juices from both ripening stages are suitable for HPU treatment because they have a solid bioactive value.

When considering the influence of HPU treatment parameters, it was found that increasing all processing parameters (e.g., amplitude, pulse, treatment duration) generally negatively affected the examined bioactive compounds. A 7 day storage had a statistically positive effect on the content of HCA and FL in HPU-treated juice samples, a negative effect on TPC and ANT, while it did not affect CT. The same trend was observed in the untreated samples, except for TPC, where 7 days of storage had a positive effect on the content.

Since a wide range of temperature changes was observed during HPU treatment of strawberry juices, the analysis showed that they were significantly correlated with almost all HPU treatment parameters (e.g., amplitude, pulses, energy, power, and frequency). Consequently, the optimal HPU parameters for these compounds were an amplitude of 25%, a pulse of 50%, and a treatment duration of 10 min. Thus, combining a suitable fruit ripening stage and HPU treatment parameters with optimal temperature variations during the treatment would be possible to obtain a high content of bioactive compounds in the juices. Therefore, HPU technology has great potential for producing functional foods based on strawberry juices.

## Figures and Tables

**Table 1:** Average values for analytical parameters in untreated samples (control).

Maturity	TPC	ANT	HCA	FL	CT
75%	93.62 ± 1.99	8.99 ± 0.13	10.56 ± 0.68	1.77 ± 0.68	70.65 ± 1.53
100%	96.18 ± 3.81	17.84 ± 0.31	16.66 ± 0.71	2.44 ± 0.82	88.75 ± 1.60

Results are expressed as the mean ± STD for control samples in mg 100 mL^−1^; TPC—total phenolic compounds; ANT—monomeric anthocyanins; HCA—hydroxycinnamic acids; FL—flavonols; CT—condensed tannins.

**Table 2:** Kruskal–Wallis test statistics for the HPU vs. control samples.

Bioactive Compound	Treatment	Mean Rank	Chi-Square	Significance
TPC	Control HPU	2.50 32.5	2.528	0.11
ANT	Control HPU	2.50 32.5	1.838	0.18
HCA	Control HPU	2.50 32.5	0.633	0.43
FL	Control HPU	2.50 32.5	1.416	0.23
CT	Control HPU	2.50 32.5	4.134	0.04

Results are expressed as the mean rank for control vs. HPU samples mg 100 mL^−1^; TPC—total phenolic compounds; ANT—monomeric anthocyanins; HCA—hydroxycinnamic acids; FL—flavonols; CT—condensed tannins.

**Table 3:** Changes in the bioactive compounds in control juice samples during storage.

Variable	n	TPC	ANT	HCA	FL	CT
Maturity	4	*p* ≤ 0.01 ^†^	*p* ≤ 0.01 ^†^	*p* ≤ 0.01 ^†^	*p* = 0.06 ^‡^	*p* ≤ 0.01 ^†^
75%	4	103.1 ± 1.26 ^a^	8.84 ± 0.09 ^b^	11.71 ± 0.24 ^b^	2.25 ± 0.55 ^a^	75.56 ± 0.91 ^b^
100%	4	90.73 ± 1.26 ^b^	17.21 ± 0.09 ^a^	17.76 ± 0.24 ^a^	4.27 ± 0.55 ^a^	86.54 ± 0.91 ^a^
Storage		*p* = 0.09 ^‡^	*p* ≤ 0.01 ^†^	*p* ≤ 0.01 ^†^	*p* = 0.02 ^†^	*p* = 0.10 ^‡^
0 days	4	94.90 ± 1.26 ^a^	13.42 ± 0.09 ^a^	13.61 ± 0.24 ^b^	2.11 ± 0.55 ^b^	79.70 ± 0.91 ^a^
7 days	4	98.94 ± 1.26 ^a^	12.63 ± 0.09 ^b^	15.86 ± 0.24 ^a^	4.41 ± 0.55 ^a^	82.39 ± 0.91 ^a^
Dataset average	8	96.92 ± 0.89	13.023 ± 0.09	14.74 ± 0.17	3.26 ± 0.39	81.05 ± 0.64

The results are expressed as the mean ± standard error in mg 100 mL^−1^. Values represented with different letters in a column are statistically different at *p* ≤ 0.05. ^†^ significant factor in multifactor analysis. ^‡^ not significant factor in multifactor analysis. TPC—total phenolic content; ANT—monomeric anthocyanins; HCA—total hydroxycinnamic acids; FL—flavonols; CT—condensed tannins.

**Table 4:** Changes in the bioactive compounds in juice samples under HPU during storage.

Variable	n	TPC	ANT	HCA	FL	CT
Maturity		*p* ≤ 0.01 ^†^	*p* ≤ 0.01 ^†^	*p* ≤ 0.01 ^†^	*p* ≤ 0.01 ^†^	*p* ≤ 0.01 ^†^
75%	64	92.61 ± 0.49 ^a^	8.22 ± 0.03 ^b^	10.66 ± 0.08 ^b^	2.06 ± 0.11 ^b^	67.23 ± 0.21 ^b^
100%	64	78.59 ± 0.49 ^b^	15.02 ± 0.03 ^a^	14.92 ± 0.08 ^a^	2.94 ± 0.11 ^a^	70.48 ± 0.21 ^a^
Amplitude		*p* ≤ 0.01 ^†^	*p* ≤ 0.01 ^†^	*p* ≤ 0.01 ^†^	*p* = 0.91 ^‡^	*p* ≤ 0.01 ^†^
25%	32	89.08 ± 0.69 ^a^	12.14 ± 0.04 ^a^	13.24 ± 0.12 ^a^	2.57 ± 0.16 ^a^	73.56 ± 0.30 ^a^
50%	32	86.22 ± 0.69 ^b^	11.48 ± 0.04 ^b^	12.83 ± 0.12 ^b^	2.54 ± 0.16 ^a^	68.87 ± 0.30 ^b^
75%	32	82.16 ± 0.69 ^c^	11.60 ± 0.04 ^c^	12.66 ± 0.12 ^b,c^	2.42 ± 0.16 ^a^	67.76 ± 0.30 ^c^
100%	32	84.94 ± 0.69 ^b^	11.27 ± 0.04 ^d^	12.44 ± 0.12 ^c^	2.47 ± 0.16 ^a^	65.23 ± 0.30 ^d^
Pulse		*p* = 0.02 ^†^	*p* ≤ 0.01 ^†^	*p* = 0.19 ^‡^	*p* = 0.05 ^†^	*p* ≤ 0.01 ^†^
50%	64	84.88 ± 0.49 ^b^	11.71 ± 0.03 ^a^	12.86 ± 0.08 ^a^	2.66 ± 0.12 ^a^	69.30 ± 0.21 ^a^
100%	64	86.32 ± 0.49 ^a^	11.53 ± 0.03 ^b^	12.72 ± 0.08 ^a^	2.34 ± 0.12 ^b^	68.41 ± 0.21 ^b^
Treatment time		*p* = 0.09 ^‡^	*p* ≤ 0.01 ^†^	*p* = 0.49 ^‡^	*p* ≤ 0.01 ^†^	*p* ≤ 0.01 ^†^
5 min	64	84.97 ± 0.49 ^a^	11.75 ± 0.03 ^a^	12.75 ± 0.08 ^a^	2.75 ± 0.12 ^a^	69.38 ± 0.21 ^a^
10 min	64	86.23 ± 0.49 ^a^	11.50 ± 0.03 ^b^	12.83 ± 0.08 ^a^	2.26 ± 0.12 ^b^	68.33 ± 0.21 ^b^
Storage		*p* ≤ 0.01 ^†^	*p* ≤ 0.01 ^†^	*p* ≤ 0.01 ^†^	*p* ≤ 0.01 ^†^	*p* = 0.54 ^‡^
0 days	64	87.25 ± 0.49 ^a^	12.03 ± 0.03 ^a^	12.41 ± 0.08 ^b^	1.81 ± 0.11 ^b^	68.76 ± 0.21 ^a^
7 days	64	83.95 ± 0.49 ^b^	11.21 ± 0.03 ^b^	13.18 ± 0.08 ^a^	3.20 ± 0.11 ^a^	68.95 ± 0.21 ^a^
Dataset average	128	85.60 ± 0.34	11.62 ± 0.02	12.79 ± 0.05	2.50 ± 0.08	68.55 ± 0.15

The results are expressed as the mean ± standard error in mg 100 mL^−1^. Values represented with different letters in a column are statistically different at *p* ≤ 0.05. ^†^ significant factor in multifactor analysis. ^‡^ not significant factor in multifactor analysis. TPC—total phenolic content; ANT—monomeric anthocyanins; HCA—total hydroxycinnamic acids; FL—flavonols; CT—condensed tannins.

**Table 5:** HPU treatment parameters.

Amplitudes (%)	Pulse (%)	Treatment Time (min)	Energy (Wh)	Power (W)	Frequency (kHz)	ΔT (°C)
25.0	50.0	10.0	2.9	40.0	23.7	4.0
25.0	50.0	5.0	1.5	42.0	23.7	8.0
25.0	50.0	10.0	3.0	42.0	23.6	12.0
50.0	50.0	10.0	3.5	78.0	23.7	14.0
50.0	50.0	5.0	1.9	71.0	23.7	15.0
50.0	50.0	10.0	3.7	71.0	23.8	16.0
25.0	50.0	5.0	1.5	45.0	23.6	17.0
25.0	100.0	5.0	3.5	44.0	23.8	18.0
66.7	66.7	6.7	3.7	78.7	23.7	19.0
50.0	100.0	5.0	6.6	90.0	23.8	21.0
37.5	75.0	7.5	4.4	52.5	23.7	22.0
25.0	100.0	5.0	3.8	38.0	23.6	23.0
75.0	50.0	5.0	2.2	85.0	23.8	25.0
100.0	50.0	7.5	3.1	88.0	23.8	26.0
75.0	50.0	10.0	4.2	85.0	23.8	29.0
75.0	100.0	5.0	10.0	133.0	23.8	31.0
75.0	50.0	10.0	4.4	74.0	23.8	33.0
75.0	75.0	10.0	7.9	76.0	23.8	34.0
100.0	100.0	5.0	11.5	177.0	23.8	35.0
50.0	100.0	5.0	6.6	93.0	23.8	36.0
100.0	100.0	5.0	11.1	166.0	23.8	37.0
50.0	100.0	10.0	12.8	90.0	23.8	46.0
75.0	100.0	7.5	13.5	129.0	23.8	49.0
100.0	100.0	10.0	20.8	177.0	23.8	50.0
75.0	100.0	10.0	16.4	128.0	23.8	51.0
100.0	100.0	10.0	19.9	176.0	23.8	54.0

**Table 6:** Pearson Correlations for HPU parameters.

	ΔT (°C)	Amplitudes (%)	Pulse (%)	Treatment Time (min)	Energy (Wh)	Power (W)	Frequency (kHz)
ΔT (°C)	1	0.59 ^†^	0.61 ^†^	0.22 ^‡^	0.85 ^†^	0.76 ^†^	0.52 ^†^
Amplitudes (%)		1	0	0	0.39 ^†^	0.79 ^†^	0.65 ^†^
Pulse (%)			1	0	0.73 ^†^	0.44 ^†^	0.13 ^‡^
Treatment time (min)				1	0.37 ^†^	−0.02 ^‡^	−0.10 ^‡^
Energy (Wh)					1	0.76 ^†^	0.31 ^‡^
Power (W)						1	0.58 ^†^
Frequency (kHz)							1

Values represented are Pearson Correlations that are statistically significant at *p* ≤ 0.05. ^†^ significant correlations; ^‡^ not significant correlations.

**Table 7:** Optimal HPU parameters for lowest ΔT and maturity with maximum mg 100 mL^−1^ of polyphenols in samples.

Analytical Variable	TPC	ANT	HCA	FL	CT
Content (mg 100 mL^−1^)	102.96	15.58	14.14	2.68	75.28
Maturity (%)	75	100	100	75	100
ΔT (°C)	4.0	35.41	36.77	4.0	4.0

TPC—total phenolic content; ANT—monomeric anthocyanins; HCA—total hydroxycinnamic acids; FL—flavonols; CT—condensed tannins.

**Table 8:** Experimental design.

Sample	Juice	Storage (Days)	Treatment	Amplitude (%)	Pulse (%)	Treatment Time (min)
1	J1	0	Control	/	/	/
2	J1	0	HPU	25	50	5
3	J1	0	HPU			10
4	J1	0	HPU		100	5
5	J1	0	HPU			10
6	J1	0	HPU	50	50	5
7	J1	0	HPU			10
8	J1	0	HPU		100	5
9	J1	0	HPU			10
10	J1	0	HPU	75	50	5
11	J1	0	HPU			10
12	J1	0	HPU		100	5
13	J1	0	HPU			10
14	J1	0	HPU	100	50	5
15	J1	0	HPU			10
16	J1	0	HPU		100	5
17	J1	0	HPU			10
18	J2	0	Control	/	/	/
19	J2	0	HPU	25	50	5
20	J2	0	HPU			10
21	J2	0	HPU		100	5
22	J2	0	HPU			10
23	J2	0	HPU	50	50	5
24	J2	0	HPU			10
25	J2	0	HPU		100	5
26	J2	0	HPU			10
27	J2	0	HPU	75	50	5
28	J2	0	HPU			10
29	J2	0	HPU		100	5
30	J2	0	HPU			10
31	J2	0	HPU	100	50	5
32	J2	0	HPU			10
33	J2	0	HPU		100	5
34	J2	0	HPU			10
35	J1	7	Control	/	/	/
36	J1	7	HPU	25	50	5
37	J1	7	HPU			10
38	J1	7	HPU		100	5
39	J1	7	HPU			10
40	J1	7	HPU	50	50	5
41	J1	7	HPU			10
42	J1	7	HPU		100	5
43	J1	7	HPU			10
44	J1	7	HPU	75	50	5
45	J1	7	HPU			10
46	J1	7	HPU		100	5
47	J1	7	HPU			10
48	J1	7	HPU	100	50	5
49	J1	7	HPU			10
50	J1	7	HPU		100	5
51	J1	7	HPU			10
52	J2	7	Control	/	/	/
53	J2	7	HPU	25	50	5
54	J2	7	HPU			10
55	J2	7	HPU		100	5
56	J2	7	HPU			10
57	J2	7	HPU	50	50	5
58	J2	7	HPU			10
59	J2	7	HPU		100	5
60	J2	7	HPU			10
61	J2	7	HPU	75	50	5
62	J2	7	HPU			10
63	J2	7	HPU		100	5
64	J2	7	HPU			10
65	J2	7	HPU	100	50	5
66	J2	7	HPU			10
67	J2	7	HPU		100	5
68	J2	7	HPU			10

J1—strawberry juice prepared from 75% ripe strawberries; J2—strawberry juice prepared from 100% ripe strawberries; Control—untreated samples.

## Data Availability

Not applicable.
